# Prokinetic and Laxative Effects of *Chrozophora tinctoria* Whole Plant Extract

**DOI:** 10.3390/molecules27072143

**Published:** 2022-03-26

**Authors:** Ayaz Ali Sher, Arshad Iqbal, Naveed Muhammad, Syed Lal Badshah, Abdul-Hamid Emwas, Mariusz Jaremko

**Affiliations:** 1 Deparment of Botany, Islamia College University, Peshawar 25120, Pakistan; ayazalisher@yahoo.com; 2 Department of Pharmacy, Abdul Wali Khan University Mardan, Mardan 23200, Pakistan; drnaveedrph@gmail.com; 3 Deparment of Chemistry, Islamia College University, Peshawar 25120, Pakistan; 4 Core Laboratories, King Abdullah University of Science and Technology (KAUST), Thuwal 23955, Saudi Arabia; abdelhamid.emwas@kaust.edu.sa; 5 Smart-Health Initiative (SHI) and Red Sea Research Center (RSRC), Division of Biological and Environmental Sciences and Engineering (BESE), King Abdullah University of Science and Technology (KAUST), Thuwal 23955, Saudi Arabia

**Keywords:** *Chrozophora tinctoria*, acetylcholinesterase inhibitor, laxative, acute toxicity, spasmogenic

## Abstract

*Chrozophora tinctoria* (Euphorbiaceae) has been used as an emetic, anthelminthic, and cathartic agent in traditional medicine. We used gas chromatography-mass spectrometry (GC-MS) to characterize the composition of ethyl acetate (EAC) and dichloromethane (DCMC) fractions from the whole *Chrozophora tinctoria* plant. EAC and DCMC fractions were evaluated for acetylcholinesterase (AChE) inhibitory activity and acute toxicity. Their effects on intestinal propulsive movement and spasmogenic activity of the gastrointestinal tract (GIT) muscle were also assessed. The compounds detected in both fractions were mostly fatty acids, with about seven compounds in EAC and 10 in DCMC. These included pharmacologically active compounds such as imipramine, used to treat depression, or hexadecanoic acid methyl ester, an antioxidant. Both EAC and DCMC fractions inhibited acetylcholinesterase (AChE) activity with IC_50_ values of 10 µg and 130 µg, respectively. Both the fractions were found to be toxic in a dose-dependent manner, inducing emesis at 0.5 g or higher and lethargy and mortality from 3–5 g upwards. Similarly, both of the fractions showed laxative activity in metronidazole- and loperamide-induced constipation models. EAC relaxed the intestinal muscle at a lower dose (1 mg/mL) than DCMC. Similarly, the EAC extract showed a significant relaxation effect (EC_50_ = 0.67 ± 0.15 mg/mL) on KCL-induced contraction in rabbit jejunum as compared to DCMC (EC_50_ = 5.04 ± 0.05 mg/kg). The present study strongly supports the folklore that this valuable plant is a cathartic agent. Further work is required to isolate and validate the bioactive compounds that act as diarrheal agents in *Chrozophora tinctoria.*

## 1. Introduction

Constipation is a chronic disorder of the gastrointestinal tract (GIT) and is defined as bowel movements of fewer than three times per week. Clinically, constipation manifests with heterogeneous symptoms including difficulty in defecation, infrequent bowel movements, hard bowel, and the feeling of incomplete defecation [1]. Constipation is equally prevalent across the globe and affects all age groups almost equally. According to one estimate, constipation is reported in 2.5–39.6% of the world’s adult population [2], while in infants to adolescents, the prevalence is in the range of 0.5–26.9% [3]. Common contributing factors to constipation include insufficient dietary fiber and fluid intake, adverse effects of medications, decreased physical activity, hypothyroidism, and colorectal cancer-induced obstruction, which can be broadly attributed to either genetic predisposition or the socioeconomic status of the patients [4]. Different drugs are applied to improve complications of constipation, including Correctol, Senna, Exlax, and Gaviscon, but with limited benefits due to the associated adverse effects and treatment costs [5,6]. The primary approach to treat constipation involves regulation of GIT motility, for which several drugs have been introduced. For instance, cisapride was among the first developed prokinetic agents but was withdrawn later due it to its effect on increasing cardiac arrhythmias [7]. Another noteworthy example is a selective 5-hydroxytrptamine-4 (5-HT_4_) receptor antagonist, tegaserod, that is currently in practice to diminish constipation despite its role in causing coronary artery diseases and myocardial infarction [8,9].

The plant kingdom has served as a remarkable resource for extracts exhibiting prokinetic activity that diminish characteristics of constipation by improving intestinal motility, defecation frequency, and stool weight. Leaf extract from the perineal plant *Ecklonia cava* showed a prominent laxative effect on the loperamide-induced constipation model in SD rates in terms of stool recovery and GIT motility. This study confirmed that the underlying principle involved facilitation of GIT hormone secretion and augmenting the downstream signaling pathway of local muscarinic acetylcholine receptors (mAChRs) [10]. Leaf extract of *Malva sylvestris* has been shown to attenuate constipation induced in male Wistar rats by increasing gastric emptying and decreasing intestinal transit time via its stimulatory effect on mAChRs [11]. In addition to intestinal transit length, a significant increase in stool frequency, weight, and water content was also observed as a result of *Ficus carica* treatment [12]. Reports such as these have encouraged many researchers to investigate medicinal plants to look for therapeutic alternatives that offer no or fewer adverse effects compared to currently available drugs.

*Chrozophora tinctoria*, commonly known as ‘dyer’s croton’ or ‘turnsole’, belongs to the family Euphorbiaceae. It is an annual plant found in Africa, Europe, and Asia. In Pakistan, it is found in tropical and temperate regions [13]. *Chrozophora* species are traditionally used to cure mouth ulcers, skin disorders such as skin burns, fever, abdominal and joint pain, jaundice, menstrual problems, wounds, GIT worms, and migraine. Moreover, *Chrozophora tinctoria* is used as an emetic and cathartic and to treat warts [14]. In Ethiopia and Senegal, the seeds and leaves of the *Chrozophora* species are used as a laxative. In Nepal, the juice obtained from its fruit is used as a remedy for cough and cold [15]. The antibacterial activity of *Chrozophora tinctoria* leaves and stem was established by earlier workers [16]. In addition, *Chrozophora tinctoria* is used for producing natural dye [17].

The presence of arabinose, fructose, glucose, raffinose, sucrose, and ribose in *Chrozophora tinctoria* has been confirmed by HPLC [18]. It has been reported that flavonoids are abundantly prevalent in almost all species of the genus *Chrozophora* [19,20,21]. HPLC analysis of the methanolic extract of the aerial parts of *Chrozophora tinctoria* reported five flavonoid glycosides. These five flavonoid glycosides are apigenin 7-O-b-d-glucopyranoside, apigenin 7-O-b-d-[(6-pcoumaroyl)]-glucopyranoside, acacetin 7-O-rutinoside, quercetin 3-O-rutinoside, and apigenin 7-O-b-d-[6-(3,4dihydroxybenzoyl)]-glucopyranoside (*Chrozophorine*) [13]. Most of these flavonoids have therapeutic properties and especially antiviral activity [22,23]. Other compounds extracted from the ethanolic extract of *Chrozophora tinctoria* are kaempferol, kaempferol 3-O-(600-a-rhamnopyranosyl)-b-glucopyranoside, kaempferol 3-O-b-glucopyranoside, two phenolics, namely, methyl gallate and gallic acid, and one steroid, namely, b-sitosterol-3-O-b-glucopyranoside. There are 35 different flavonoids found in various species of the genus *Chrozophora,* which have anti-proliferative, antioxidant, antimicrobial, antipyretic, and anti-nociceptive properties [24]. The leaves of *C. tinctoria* possess antidiabetic and hepatoprotective properties [25]. Rutin present in *Chrozophora* has the ability to promote bone cell growth [26]. Here, we used gas chromatography-mass spectrometry to investigate the phytochemical profile of fractions taken from *Chrozophora tinctoria* and evaluate their laxative activity as novel treatment options for constipation.

## 2. Results

### 2.1. GC-MS

#### 2.1.1. Composition of EAC

**Figure 1:**
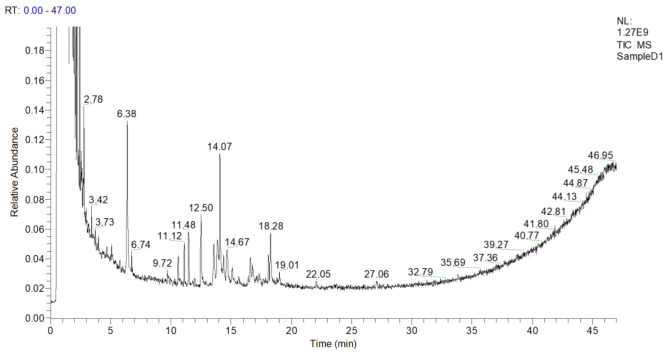
GC-MS chromatogram of the ethylacetate fraction of *Chrozophora tinctoria*. The numbers show the retention times of various compounds.

About seven compounds were tentatively identified through GC-MS. The GC-MS chromatogram demonstrated seven phytochemical constituents, as presented in Figure 1, Table 1 and Supplementary Table S1. These peaks were matched with the database of known components present in the GC-MS library. Compounds having a similarity index of more than 600 were recorded. GC-MS analysis of EAC showed the presence of many bioactive compounds at different retention times (min).

#### 2.1.2. Composition of DCMC

**Figure 2:**
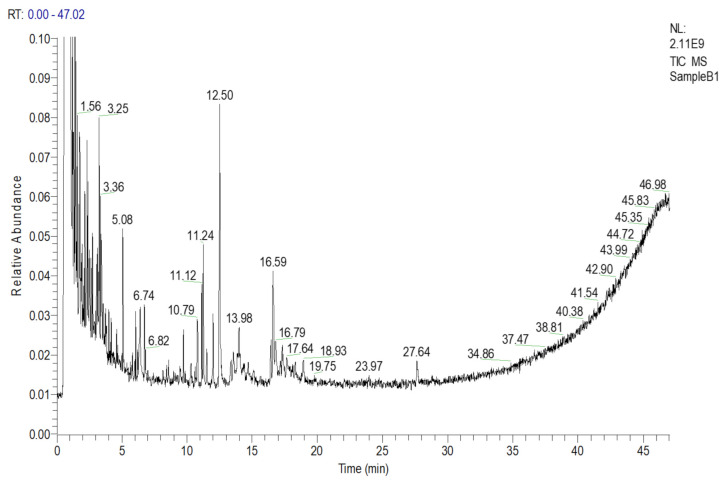
GC-MS chromatogram of the dichloromethane fraction (DCMC) of *Chrozophora tinctoria.* The numbers inside the chromatogram are the retention times of the compounds.

Similarly, a total of 10 chemical compounds from the DCMC fraction were tentatively identified with a similarity index of more than 600, after matching the different retention times with the library, as shown in Figure 2 and Table 2.

### 2.2. Acetylcholinesterase Inhibitory Activity

#### Effect of EAC and DCMC

The EAC and DCMC fractions demonstrated AChE inhibition at various concentrations, as presented in Table 3. The percent inhibition of 1000 µg/mL EAC was 93.33 ± 1.53. The inhibitory concentration (IC_50_) of the EAC fraction was 10 µg/mL, showing good AChE inhibitory potential as compared to galantamine (IC_50_ = 5 µg/mL). Similarly, 1000 µg/mL of DCMC showed good enzyme inhibitory activity, with AChE inhibition of 68.33%.

### 2.3. Acute Toxicity

#### 2.3.1. Acute Toxicity of EAC and DCMC

The acute toxicity parameters of EAC and DCMC are shown in Table 4. Doses up to 0.3 g/kg were safe and without any toxic symptoms; however, at a concentration of 0.5 g/kg and upward, emesis and diarrhea were noticed. Concentrations of 4 and 5 g/kg were found to be emetic and diarrheal with 25.0% mortality.

#### 2.3.2. Effect on Metronidazole-Induced Constipation

EAC and DCMC were both prokinetic against metronidazole-induced constipation, as presented in Table 5 and Table 6, respectively. The tested groups treated with EAC and metronidazole (Metro) demonstrated that the prokinetic effects of EAC were significantly (*p* < 0.001) inhibited, as shown in Table 6. The total number of stools, number of wet stools, and percent of wet stool were significantly (*p* < 0.001) diminished by metronidazole-treated groups as compared to EAC- and DW-tested groups. 

#### 2.3.3. Effect of Loperamide-Induced Constipation 

The prokinetic activity of EAC and DCMC was significantly attenuated by loperamide (4 mg/kg), as presented in Table 7 and Table 8, respectively. Thus, the percent of wet stool with EAC doses of 1, 2, and 3 g/kg was significantly (*p* < 0.001) reduced from 63.03, 77.64, and 80.55% to 20.16, 43.30, and 62.02%, respectively. The latency time was also significantly (*p* < 0.001) delayed by the loperamide. A similar attenuation in the prokinetic effect of DCMC was observed against loperamide-induced constipation. 

### 2.4. Gastrointestinal Motility (Charcoal Meal Method)

The charcoal meal transit of EAC and DCMC is presented in Table 9. The charcoal movement of EAC at 25, 50. and 100 mg/kg was 33.41, 53.97, and 75.20%, respectively. The effect of DCMC at the same doses was 31.50, 41.20, and 51.84%, respectively. 

### 2.5. Spasmolytic Activity

**Figure 3:**
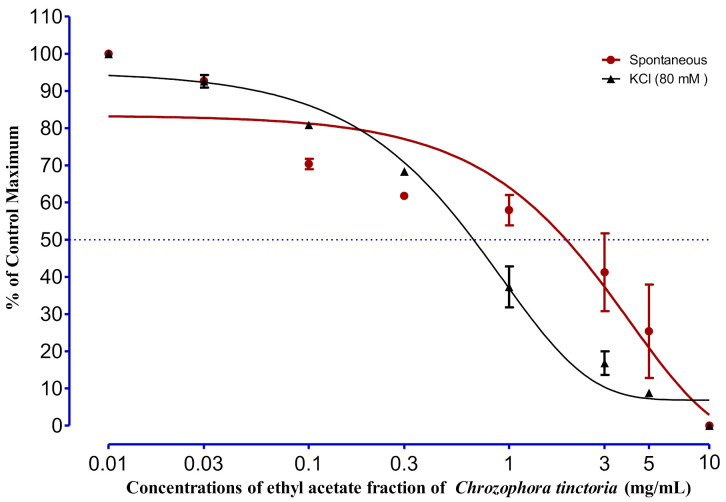
The effect of EAC on spontaneous and KCL-induced (80 mM) contraction of rabbit jejunum. Rabbit jejunum muscle was relaxed in a dose-dependent manner. EC_50_ values were calculated from curve fitting in GraphPad prism 6.01. Each point represents the mean ± SEM of grouped data.

**Figure 4:**
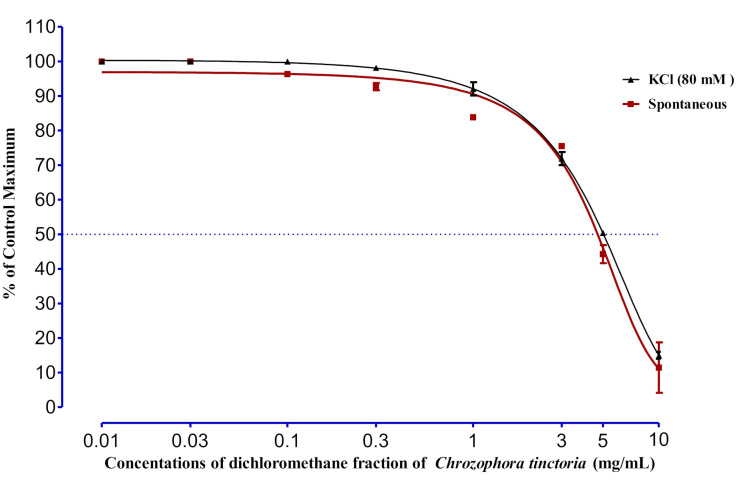
The effect of DCMC on spontaneous and KCL-induced (80 mM) contraction of rabbit jejunum. Rabbit jejunum muscle was relaxed in a dose-dependent manner. EC_50_ values were calculated from curve fitting in GraphPad prism 6.01. Each point represents the mean ± SEM of grouped data.

Significant relaxations in the spontaneous responses were noted at a concentration of 1 mg/mL; however, complete relaxation in spontaneous responses was detected at a concentration of 10 mg/mL with EC_50_ of 2.11 ± 1.20 mg/mL. In the case of KCL-induced (80 mM) contraction, the relaxing effect of EAC started gradually from 0.1 and 0.3 mg and showed a complete relaxing effect at 10 mg/mL, with an EC_50_ value of 0.67 ± 0.15 mg/mL (Figure 3). The effect of DCMC on spontaneous rabbit jejunal preparation is described in Figure 4. Different concentrations were tested, as shown in the figure; significant results were shown by 3, 5, and 10 mg/kg, with EC_50_ equal to 4.71 ± 0.26 mg/kg. In the same way, the 3-, 5-, and 10-mg/kg concentrations of DCMC had a relaxing effect on KCL-induced (80 mM) contraction in rabbit’s jejunum, with an EC_50_ value of 5.04 ± 0.05 mg/kg.

## 3. Discussion

Constipation is a worldwide problem that is equally prevalent in all age groups. Different pharmacological and non-pharmacological paradigms are available for relieving the symptoms of constipation. Among the pharmacological approaches, various allopathic and herbal-based therapeutic options are practiced. Even though the allopathic system offers various synthetic and semisynthetic molecules for the treatment of constipation, numerous side effects are attributed to those approaches. Therefore, the search for a safe, effective, and affordable therapeutic agent for constipation is a challenge to current medical science. In this study, the prokinetic effect of *Chrozophora tinctoria* fractions was investigated to establish pharmacological grounds for the potential use of these fractions in constipation therapy. 

AChE is a key enzyme found in the blood and nervous system, mediating various important physiological functions. The principal role of this enzyme is the cessation of nerve impulses at cholinergic synapses by breaking down the neurotransmitter acetylcholine into choline and acetic acid. The inhibition of acetylcholinesterase is a promising strategy against Parkinson disease, myasthenia gravis, ataxia, senile dementia, and Alzheimer disease [27]. Phytochemicals derived from other plants have shown inhibitory action against acetylcholinesterase [28]. The EAC fraction of *Chrozophora tinctoria* significantly inhibited AchE, with an IC_50_ of 10 µg/mL. AChE inhibition implies blockade of ACh degradation, resulting in a higher concentration of ACh. This availability of ACh will result in a prolonged interaction with the muscarinic and nicotinic receptors, maintaining the impulse. Ach is bioavailable in various conformational isomers (gauche and anti-gauche forms), which is the reason behind its interaction with both cholinergic receptors. The cholinergic response will promote diarrhea through its intracellular signaling pathways. The laxative effect of *Chrozophora tinctoria* might be related to the cholinergic action of the tested fractions. The laxative activity of both fractions was studied in metronidazole- (7 mg/kg) and loperamide hydrochloride- (4 mg/kg) induced constipated pigeons. The prokinetic potential of the tested fractions was attenuated by loperamide more significantly than metronidazole. The prokinetic effect of the *Chrozophora tinctoria* fractions was further confirmed by the charcoal meal propulsion in the intestine of pigeons. Both of the fractions demonstrated significant (*p* ≤ 0.05) propulsion of charcoal in the animal intestine. 

Contraction of intestinal smooth muscle results from periodic depolarization. Thus, depolarization in intestinal smooth muscle is usually attributed to the release of calcium ions, either from intracellular stores or through the influx of calcium to the inside of the tissue through voltage-gated calcium channels. KCL in high doses is considered a depolarizer, which may trigger cellular calcium influx. Therefore, a relaxing effect in KCL-induced contraction usually indicates that the test substance induces calcium channel blockade [29]. This experiment was conducted to investigate the direct effects of EAC and DCMC on rabbit jejunum. The antispasmodic effect of EAC on the smooth muscles of the jejunum was detected at a concentration of 1 mg/mL. Similarly, the spasm induced in rabbit’s jejunum by KCL (80 mM) was relaxed by EAC completely at 10 mg/mL. Similarly, at a concentration of 3 mg/kg the DCMC exhibited a relaxing effect on KCL-induced (80 mM) contraction in rabbit’s jejunum with an EC_50_ value of 5.04 ± 0.05 mg/kg. It was deduced that the relaxing effect with EC_50_ 2.11 ± 1.20 mg/mL of EAC and an EC_50_ value of 5.04 ± 0.05 mg/kg of DCMC may have been due to the inhibition of voltage-gated calcium channels. Furthermore, it also implies that EAC was more potent than DCMC. The tested fractions were also investigated for acute toxicity on pigeons. The animal model used in this study is considered a paradigm for the induction of emesis [30], as compared to other emesis models, such as mice and rats, which are usually not as responsive. The acute toxicity study confirmed that the plant fractions used in this study induce emesis, a potential side effect of EAC and DCMC. Strikingly, the tested plant fractions are also anthelmintic as dead worms were observed in the stool passed by animals used in this study. The spasmolytic effect can be a contributing factor to the anthelmintic action of *Chrozophora tinctoria.* A further molecular-level study is needed to elaborate on the underlying mechanisms of the EAC and DCMC fractions.

## 4. Materials and Methods

### 4.1. Chemicals and Solvents

The chemicals/drugs used in the present study were Maxolon^®^ (Valeant Pharmaceutical International, Inc., Karachi, Pakistan), Gravinate^®^ (The Searle company (PVT) LTD, Karachi, Pakistan), Imodium^®^ (ASPIN Pharma PVT. LTD, Karachi, Pakistan), Flagyl^®^ (Sanofi Aventis (PVT) LTD, Pakistan), Copper Sulphate (Nenza pharmaceuticals (PVT) LTD, Peshawar, KPK, Pakistan), Cisplatin (Pfizer Laboratories LTD, Karachi, Pakistan), Magnesium Sulphate (Nenza Pharmaceuticals (PVT) LTD, Pakistan), castor oil (Karachi Pharmaceuticals Laboratory, Karachi, Pakistan), gum acacia (Shreeji Pharma International), normal saline (Shahzeb Pharmaceutical, Haripur, KPK, Pakistan), methanol (Master Chemical Supplier, Karachi, Pakistan), and ethyl acetate (Master Chemical Supplier, Karachi, Pakistan).

### 4.2. Instruments 

A rotary evaporator (Model RE-111, Bochi, Switzerland), 5-cc, 3-cc, and 1-cc syringes (Shifa disposable syringe), a drip set (Shifa drip set, Pakistan), water bath (Thermostatic controlled-STD/GMP), magnetic stirrer (H3760-S Digital magnetic stirrer), and analytical balance (Shimadzo analytical balance) were used.

### 4.3. Plant Collection and Identification

Mature *Chrozophora tinctoria* was collected from the Mohmand Agency, Khyber Pakhtunkhwa, Pakistan, and was authenticated by Dr. Sher Wali, Assistant Professor, Department of Botany, Islamia College Peshawar. The identified plant specimen was given a voucher number (CT-Bot-11082017) and deposited in the Herbarium, Department of Botany, Islamia College Peshawar. The collected plant was washed with clean water to remove dirt, and was dried at room temperature by spreading it in a single layer on blotting paper. After drying, the plant was cut into fine pieces and was powdered using an electric grinder. 

### 4.4. Extraction and Fractionation

Extraction and fractionation of the desired fractions were performed according to the literature with some modifications [31,32]. Maceration in methanol was carried out for a total of 10 kg of powdered plant with random shaking for several days followed by filtration through Whatmann filter paper No 1. The filtrate was concentrated by a rotary evaporator to obtain a crude methanolic extract. The dried crude methanolic extract was dissolved in distilled water and then n-hexane was added. After shaking in a separating funnel, the n-hexane fraction was obtained and concentrated by a rotary evaporator. Similarly, dichloromethane, ethyl acetate, and n-butanol fractions were obtained. This method is called solvent–solvent fractionation. Their order of fractionation was from n-hexane then dichloromethane, ethyl acetate, and n-butanol, respectively. DCMC and EAC fractions were used for further activities [31,32].

### 4.5. Gas Chromatography-Mass Spectrometry (GC-MS)

To identify different phytochemicals present in EAC and DCMC fractions, the samples were subjected to GC-MS. The samples were checked using a Thermo Scientific (DSQ-II) GC. The GC device was furnished with a 30-m-long TR-5MS capillary column and a 0.25-µm-thick film and had 0.25 mm of internal diameter. Helium was used as a carrier gas with a flow rate of 1 mL/min. The injection device was run in a split mode at 250 °C. The sample was injected, 1 µL at a time, with an initial oven temperature of 50 °C that was maintained for 2 min followed by gradually elevating the temperature to 150 °C at a rate of 8 °C/min. Finally, the temperature was raised to 300 °C at a rate of 15 °C/min and maintained for 5 min [33,34].

### 4.6. Pharmacological Activities

#### 4.6.1. Animals

##### Pigeons

Healthy pigeons were defined as those with normal stools (non-diarrheal and non-emetic), that were non-lethargic, and had no weight loss, no nasal dropping, no shedding or feather ruffling, and usual flying movement with regular feeding. Mature pigeons of both sexes were selected with weights in the range of 240–380 g. Animals were provided with standard food (locally available food; millet + wheat grains), fresh water, and a light/dark cycle for 12/12 h for 5–7 days. On the day of the experiment, the pigeons were weighed and health was assessed. Pigeons observed as unhealthy were removed from the experiment. After selection, animals were placed in separate cages to get individual data of each group [35]. Pigeons were held gently, and fractions were given orally by a feeding tube with the help of an assistant. 

##### Rabbits

Healthy rabbits were defined as being active and alert with no wet nose, runny eyes, scabby ears, or sore spots on their feet, with normal feeding and feces. Mature male rabbits weighing 1.5–3 kg were selected for the experiment. Rabbits were served with standard food and fresh water for several days. On the day of the experiment, the rabbits were examined again and the healthy ones were subjected to cervical dislocation [36]. The abdomen of the animal was opened after cervical dislocation and about 1.5–2.5-cm slices of jejunum were detached and placed in Tyrode solution in Petri dishes with a continuous supply of carbogen gas (95% O_2_ and 5% CO_2_). The jejunum was then used for further experiments.

#### 4.6.2. In Vitro Experiments

##### Acetylcholinesterase Assay

Increasing concentrations of EAC (125, 250, 500, 1000 µg/mL) and DCMC fractions were tested for AChE using a spectrophotometer according to the method of Ellman [37]. This method for assaying thiols is based on the principle that acetylthiocholine iodide is hydrolyzed by the respective enzyme-producing thiocholine, which reacts with 5,5′-dithiobis-2-nitrobenzoic acid (DTNB) (Ellman’s reagent). The final products of the second reaction are 5-thio-2 nitrobenzoate and 2-nitrobenzoate-5-mercaptothiocholine. The absorption of the former product is measured by a spectrophotometer (412 nm). The positive control, galantamine, was prepared in the same concentrations as EAC. The test and control solutions were incubated at 37 °C for 20 min. The enzyme inhibition was calculated from the absorption rate with a change in time [37,38].

The percent inhibition was calculated as

Enzyme inhibition (%) = 100 − percent enzyme activity

Percent enzyme activity (%) = 100 × V/V_max_

where (V_max_) is an enzyme activity in the absence of an inhibitor.

#### 4.6.3. In Vivo Experiments

##### Acute Toxicity

Acute toxicity of EAC and DCMC was established according to an available protocol with slight modifications [39]. The pigeons were divided into two groups (n = 8). One group received a fraction while the negative control group received distilled water only (6 mL/kg, PO). Each fraction was administered orally with 0.3, 0.5, 1, 2, 3, 4, and 5 g/kg as a single dose using a feeding tube to different groups. All the animals were observed for toxic symptoms, i.e., diarrhea, emesis, lethargy, and motility, for about 72 h [39]. 

##### Laxative Activity

Pigeons were divided into eight groups (n = 8). A white, plastic base was provided in the cages for stool collection and examination. Groups 1, 2, and 3 were administered distilled water and 1-g, 2-g, and 3-g doses of the fraction, respectively. Group 4 was given distilled water (6 mL/kg) and group 5, castor oil (6 mL/kg P.O). Groups 6, 7, and 8 were given 1 g, 2 g, and 3 g of the fractions, respectively. Constipation was induced in all the groups except groups 1, 2, and 3s by administering metronidazole (7 mg/kg) and loperamide hydrochloride (6 mg/kg). After 30 min, the first stool time/latency time (min), number of stools, number of wet stools, and weight of stool (g) were recorded and the percent effect was calculated as follows:

$$\mathrm{Percent}\;\mathrm{inhibition}=\frac{\mathrm{Number}\;\mathrm{of}\;\mathrm{wet}\;\mathrm{stools}\;\mathrm{of}\;\mathrm{individual}}{\mathrm{Total}\;\mathrm{number}\;\mathrm{of}\;\mathrm{stools}\;\mathrm{of}\;\mathrm{individual}}\times 100$$

##### Charcoal Meal Treatment

Pigeons were divided into five groups (n = 8). Constipation was induced in all the groups by loperamide hydrochloride. Group 1 was given distilled water (2 mL) and group 2 was served with castor oil while groups 3, 4, and 5 received fractions with a concentration of 25, 50, and 100 mg/kg, respectively. After 30 min, 2 mL of charcoal meal (a solution of 10% charcoal and 5% gum acacia) was given orally to each pigeon. The animals were then provided food and water, and, after 30 min, they were sacrificed. Then, the whole intestine, starting from the pylorus region up to the ileocecal junction, was removed from the pigeons and was placed on white paper parallel to a ruler. The distance travelled by the charcoal marker was measured and expressed as percent intestinal transit [40]. The percent effect was calculated as follows:

$$\mathrm{Percent}\;\mathrm{intestinal}\;\mathrm{transit}=\frac{\mathrm{Distance}\;\mathrm{travel}\;\mathrm{by}\;\mathrm{charcoal}}{\mathrm{The}\;\mathrm{total}\;\mathrm{length}\;\mathrm{of}\;\mathrm{intestine}}\times 100$$

##### Spasmolytic Activity

The spasmolytic activity of the fractions was assessed according to earlier reported studies [36]. Briefly, the abdomen of the rabbit was opened after cervical dislocation and about 1.5–2.5-cm slices of jejunum were detached and placed in Tyrode solution in Petri dishes with a continuous supply of carbogen gas (95% O_2_ and 5% CO_2_). The mesentery was removed from the separated jejunum tissue and was fixed in the organ bath containing Tyrode solution at a retained temperature of 37 ± 1 °C. After this, the tissue was stabilized in the organ bath for about 20–30 min. The stable tissue with spontaneous response was taken as a baseline control, which is a positive control. The relaxing effect of the tested plant extract was compared. 

Then, the effect of the fractions on spontaneous activity of the jejunum preparation was carried out at different concentrations, i.e., 0.01, 0.03, 0.1, 0.3, 3.0, 5.0, and 10 mg/mL, at an interval of 1 to 2 min in a cumulative manner, and the effect was noted. The effect of the fractions was also tested against KCL-induced contraction.

### 4.7. Ethical Approval

The study was approved by the ethical board of the Department of Pharmacy, Abdul Wali Khan University, Mardan, Pakistan. The ethical approval no. is EC/PhM/AWKUM-871D. 

### 4.8. Statistical Analysis

Data are expressed as the mean. One-way ANOVA followed by Dunnett’s test was applied. The concentration–response curve was plotted using Graph Pad Prism for Windows 6.0 (Graph Pad Software, San Diego, CA, USA).

## 5. Conclusions

Fractions prepared from *Chrozophora tinctoria* exhibited prominent laxative and spasmogenic activities. The laxative effects were observed by AChE inhibition and the charcoal meal assay. Possible side effects from the plant extracts are nausea and vomiting. The current study strongly supports the folklore of *Chrozophora tinctoria* as a potential cathartic to be used in constipation treatment, although further molecular studies are needed to establish the underlying pharmacology.

## Figures and Tables

**Table 1:** Bioactive compounds identified in the ethylacetate fraction (EAC) through GC-MS.

Peak No.	Retention Time (min)	SI	RSI	Area %	Probability	Compound Name	Formula	Molecular Weight	Library
1	2.78	783	980	0.01	93.57	Imipramine	C_19_H_24_N_2_	280	nist_msms
2	6.38	905	914	0.06	76.55	Hexadecanoic acid, methyl ester	C_17_H_34_O_2_	270	replib
3	11.12	684	870	0.01	22.81	3,7,11,15-Tetramethyl-2-hexadecen-1-ol	C_20_H_40_O	296	MAINLIB
4	12.50	865	889	0.02	48.69	Pentadecanoic acid, 14-methyl-, methyl ester	C_17_H_34_O_2_	270	MAINLIB
5	14.07	846	866	0.07	80.32	Hexadecanoic acid, ethyl ester	C_18_H_36_O_2_	284	MAINLIB
6	14.67	612	652	0.01	45.49	Nonanoic acid, 9-(0-propylphenyl)- methyl ester	C_19_H_30_O_2_	290	MAINLIB
7	18.28	728	749	0.03	12.14	9,12,15-Octadecatrienoic acid, ethyl ester, (Z,Z,Z)-	C_20_H_34_O_2_	306	replib

**Table 2:** Bioactive compounds identified in DCMC through GC-MS.

Peak No.	Retention Time (min)	SI	RSI	Area %	Probability	Compound Name	Formula	Molecular Weight	Library
1	1.56	742	812	0.06	51.92	Hydroperoxide, 1-ethyl butyl	C_6_H_14_O_2_	118	MAINLIB
2	3.25	657	722	0.04	40.54	Silane, chlorodiisopropylmethyl-	C_7_H_17_ClSi	164	MAINLIB
3	5.08	712	891	0.01	4.42	1-Hexadecene	C_16_H_32_	224	MAINLIB
4	10.79	686	757	0.01	26.35	3,7,11,15-Tetramethyl-2-hexadecen-1-ol	C_20_H_40_O	296	MAINLIB
5	12.50	912	919	0.06	72.93	Hexadecanoic acid, methyl ester	C_17_H_34_0_2_	270	MAINLIB
6	13.98	728	893	0.03	6.03	1-Eicosanol	C_20_H_42_O	298	replib
7	16.59	775	791	0.05	8.39	10-Octadecanoic acid, methyl ester	C_19_H_36_O_2_	296	MAINLIB
8	17.64	674	714	0.01	25.03	9,12,15-Octadecatrienoic acid, 2,3-bis[(trimethylsilyl)oxy]propyl ester, (Z,Z,Z)-	C_27_H_52_O_4_Si_2_	496	replib
9	18.93	651	675	0.01	6.21	17-Pentatriacontene	C_35_H_70_	490	MAINLIB
10	27.64	727	899	0.01	41.12	1,2-Benzenedicarboxylic acid, diisooctyl ester	C_24_H_38_O_4_	390	replib

**Table 3:** Percent inhibition of acetylcholinesterase by EAC.

Compound Name/Plant Name	Fraction	Concentration (µg/mL)	% AChE Inhibition	IC_50_
Galantamine	Standard	1000	95.67 ± 2.52	5.0
		500	87.33 ± 2.52	
		250	82.67 ± 3.06	
		125	77.00 ± 3.00	
*Chrozophora tinctoria*	EAC	1000	93.33 ± 1.53	10
		500	87.33 ± 3.06	
		250	80.67 ± 2.08	
		125	73.00 ± 4.58	
*Chrozophora* *tinctoria*	DCMC	1000	68.33 ± 2.52	130
		500	63.00 ± 3.61	
		250	57.67 ± 2.31	
		125	48.67 ± 2.08	

Values are expressed as mean ± SEM. Statistical significance was determined using IC_50_ values through Biostata software.

**Table 4:** Acute toxicity of the ethyl acetate and dichloromethane fractions of *Chrozophora tinctoria* in pigeons.

Sample	Dose (g).(mL)/kg	Emesis	Diarrhea	Lethargy	Mortality (%)
		Total Number of Vomits	Total Number of Wet Stools		
Distilled water	6	0.00 ± 0.00	0.00 ± 0.00	-	0.00 ± 0.00
EAC	0.3	0.00 ± 0.00	0.00 ± 0.00	-	0.00 ± 0.00
	0.5	2.00 ± 0.82	0.00 ± 0.00	-	0.00 ± 0.00
	1	6.00 ± 2.65 *	8.33 ± 4.51	-	0.00 ± 0.00
	2	6.67 ± 2.52 *	10.00 ± 3.00 *	-	0.00 ± 0.00
	3	8.00 ± 2.00 ***	10.67 ± 3.06 **	Less	0.00 ± 0.00
	4	9.33 ± 1.53 ***	12.33 ± 2.52 ***	More	25.00
	5	11.00 ± 3.00 ***	14.00 ± 4.00 ***	More	25.00
DCMC	0.3	0.00 ± 0.00	0.00 ± 0.00	-	0.00 ± 0.00
	0.5	2.33 ± 0.58	0.00 ± 0.00	-	0.00 ± 0.00
	1	6.33 ± 1.53 *	6.00 ± 3.00	-	0.00 ± 0.00
	2	7.00 ± 1.73 **	8.67 ± 2.08	-	0.00 ± 0.00
	3	9.67 ± 2.08 ***	9.33 ± 2.52 *	Less	0.00 ± 0.00
	4	10.00 ± 2.65 ***	9.67 ± 4.04 *	More	25.00
	5	11.33 ± 2.31 ***	11.00 ± 1.00 **	Most	25.00

Values are expressed as mean ± SEM. Statistical significance was determined with one-way ANOVA followed by Tukey’s multiple comparison test (using Graph Pad Prism 6.01 software); * *p* ≤ 0.05 was considered statistically significant (** *p* ≤ 0.01, *** *p* ≤ 0.001).

**Table 5:** Diarrheal/laxative activity of EAC.

Samples	Dose g/kg mL/kg (PO)	First Stool/Latency Time (Minutes)	Total Number of Stools	Number of Wet Stools	Weight of Stool (Grams)	Percent Wet Stool (%)
EAC + DW	1	28.00 ± 4.32	17.25 ± 3.30	8.75 ± 2.50	8.25 ± 1.71	50.41 ± 8.33
	2	25.00± 1.83	19.00 ± 2.94	10.25 ± 1.71	10.50 ± 2.65	53.98 ± 4.89
	3	22.00 ± 1.83	21.00 ± 2.16	15.75 ± 1.89	12.25 ± 2.75	72.65 ± 6.64
Metronidazole (7 mg/kg) was administered (PO) 30 min before fraction/distilled water/castor oil						
Distilled water (-ve control)	6	73.33 ± 2.52	9.33 ± 2.52	0.00 ± 0.00	8.27 ± 1.96	0.00 ± 0.0
Castor oil (+ve control)	6	20.33 ± 2.52 ***	22.00 ± 2.00 ***	18.33 ± 1.53 ***	18.67 ± 2.36 ***	83.43 ± 2.23 ***
EAC + Metro	1	35.33 ± 3.06 ***	13.00 ± 3.00	5.00 ± 1.00 **	8.37 ± 1.03	38.65 ± 1.26 ***
	2	35.00 ± 2.00 ***	16.67 ± 1.15 ***	6.00 ± 2.00 ***	10.17 ± 1.96	35.65 ± 9.85 ***
	3	31.00 ± 1.73 ***	18.00 ± 1.00	6.00 ± 2.00 ***	10.90 ± 1.80	63.43 ± 3.33 ***

Data are presented as the mean ± SEM; one-way ANOVA followed by Dunnett’s test was applied to determine significance; *p* ≤ 0.05 was considered as significant (** *p* ≤ 0.01; *** *p* ≤ 0.001). EAC, ethyl acetate fraction of *Chrozophora tinctoria*; Metro, metronidazole; DW, distilled water; PO, per orally.

**Table 6:** Diarrheal/laxative activity of DCMC.

Samples	Dose g/kg mL/kg (PO)	First Stool/Latency Time (Minutes)	Total Number of Stools	Number of Wet Stools	Weight of Stool (Grams)	Percent Wet Stool (%)
DCMC + DW	1	30.00 ± 2.94	16.25 ± 1.71	5.25 ± 1.71	10.10 ± 1.34	31.79 ± 8.13
	2	28.75 ± 4.27	18.25 ± 2.06	7.00 ± 1.63	13.00 ± 2.16	38.31 ± 7.34
	3	27.50 ± 3.42	19.75 ± 2.99	9.00 ± 2.16	14.78 ± 3.03	45.18 ± 6.29
Metronidazole (7 mg/kg) was administered (PO) 30 min before fraction/distlled water/castor oil						
Distilled water (-ve control)	6	73.33 ± 2.52	9.33 ± 2.52	0.00 ± 0.00	8.27 ± 1.96	0.00 ± 0.00
Castor oil (+ ve control)	6	20.33 ± 2.52 ***	22.00 ± 2.00 ***	18.33 ± 1.53 ***	18.67 ± 2.36 ***	83.43 ± 2.23 ***
DCMC + Metro	1	38.00 ± 3.00 ***	12.00 ± 2.65	3.00 ± 1.00	7.30 ± 1.21	24.64 ± 4.03 ***
	2	39.67 ± 3.06 ***	15.33 ± 1.53 **	5.33 ± 2.52 **	9.73 ± 1.42	33.93 ± 12.83 ***
	3	39.33 ± 1.53 ***	15.00 ± 1.73	5.00 ± 1.00 **	9.07 ± 1.17	33.33 ± 5.56 ***

Data are presented as mean ± SEM; one-way ANOVA followed by Dunnett’s test was done to determine statistical significance where *p* ≤ 0.05 was considered as statistically significant (** *p* ≤ 0.01; *** *p* ≤ 0.001). DCMC, dichloromethane fraction of *Chrozophora tinctoria*; Metro, metronidazole; DW, distilled water; PO, per orally/by orally.

**Table 7:** Diarrheal/laxative activity of EAC.

Samples	Dose g/kg mL/kg (PO)	First Stool/Latency Time (Minutes)	Total Number of Stools	Number of Wet Stools	Weight of Stool (Grams)	Percent Wet Stool (%)
EAC + DW	1	23.25 ± 2.50	14.75 ± 3.59	09 ± 2.16	10.05 ± 2.87	63.03 ± 13.97
	2	21.5 ± 1.73	15.75 ± 1.71	12 ± 1.63	11.33 ± 3.21	77.64 ± 18.26
	3	19.75 ± 2.22	16 ± 2.45	13 ± 2.94	13 ± 2.45	80.55 ± 7.97
Loperamide hydrochloride (4 mg/kg) was administered (PO) 30 min before fractions/distilled water/castor oil.						
Distilled water (-ve control)	6	70.33 ± 2.52	8.33 ± 2.52	0.00 ± 0.00	7.50 ± 2.29	0.00 ± 0.00
Castor oil (+ ve control)	6	17.33 ± 2.52 ***	16.67 ± 2.08 **	14.33 ± 1.53 ***	15.07 ± 1.27 ***	89.74 ± 2.78 ***
EAC + Lopr	1	41.33 ± 3.21 ***	11.00 ± 2.65	2.00 ± 1.00	8.43 ± 1.81	20.16 ± 13.10 ***
	2	40.33 ± 1.53 ***	11.67 ± 3.06	5.00 ± 1.00 *	9.23 ± 1.00	43.30 ± 2.90 ***
	3	30.33 ± 1.53 ***	9.67 ± 1.53	6.00 ± 1.00 **	9.20 ± 1.05	62.04 ± 1.86 ***

Data are represented as mean ± SEM. The data were analyzed by one-way ANOVA followed by Dunnett’s test using GraphPad prism version 6.01; *p* ≤ 0.05 was considered as significant (* *p* ≤ 0.05; ** *p* ≤ 0.01; *** *p* ≤ 0.001). EAC, ethyl acetate fraction of *Chrozophora tinctoria*; Lopr, loperamide hydrochloride; DW, distilled water, PO, per orally/by orally.

**Table 8:** Diarrheal/laxative activity of DCMC.

Samples	Dose g/kg mL/kg (PO)	First Stool/Latency Time (Minutes)	Total Number of Stools	Number of Wet Stools	Weight of Stool (Grams)	Percent Wet Stool (%)
DCMC + DW	1	33.25 ± 3.50	15.00 ± 3.56	5.00 ± 2.83	8.62 ± 2.44	32.68 ± 12.27
	2	30.25 ± 3.30	16.75 ± 2.22	7.25 ± 2.50	10.25 ± 2.55	42.16 ± 10.00
	3	28.25 ± 2.06	19.5 ± 2.52	9.25 ± 2.63	10.33 ± 1.79	46.79 ± 7.03
Loperamide hydrochloride (4 mg) was administered (PO) 30 min before of fractions/distilled water/castor oil.						
Distilled Water (-ve control)	6	70.33 ± 2.52	8.33 ± 2.52	0.00 ± 00	7.50 ± 2.29	0.00 ± 00
Castor oil (+ ve control)	6	17.33 ± 2.52 ***	16.00 ± 2.00 **	14.33 ± 1.53 ***	15.07 ± 1.27 ***	89.74 ± 2.78 ***
DCMC + Lopr	1	40.00 ± 2.65 ***	10.33 ± 2.08	1.67 ± 0.58	8.20 ± 1.30	16.92 ± 7.95 **
	2	37.00 ± 2.00 ***	11.67 ± 1.53	3.67 ± 0.58	8.50 ± 0.62	30.00 ± 3.34 ***
	3	33.67 ± 3.06 ***	10.00 ± 1.00	6.00 ± 1.00 **	8.30 ± 0.79	35.45 ± 5.06 ***

Data are represented as mean ± SEM. The data were analyzed by one-way ANOVA followed by Dunnett’s test using GraphPad prism 6.01 version; *p* ≤ 0.05 was considered as significant (** *p* ≤ 0.01; *** *p* ≤ 0.001). DCMC, dichloromethane fraction of *Chrozophora tinctoria*; Lopr, loperamide hydrochloride; DW, distilled water; PO, per orally/by orally.

**Table 9:** Percent intestinal transit of EAC and DCMC in the pigeons.

Treatment	Dose mg/Kg (PO)	Total Length of Intestine (cm)	Total Distance Travelled by Charcoal Meal (cm)	% of Intestinal Transit
Castor oil	4 mL	88.9 ± 5.1	77.7 ± 5.1	87.60 ± 5.8
Immodium	6	92.3 ± 3.9	30.3 ± 6.8	32.7 ± 6.7
DW	4 mL	90.9 ± 2.7	37.3 ± 2.8	41.15 ± 4.3
EAC	25	91.4 ± 2.3	30.5 ± 1.9	33.41 ± 2.5
	50	71.9 ± 1.6	38.7 ± 1.3	53.97 ± 2.7
	100	73.7 ± 3.1	55.3 ± 1.9	75.2 ± 4.9
DCMC	25	66.0 ± 2.0	20.8 ± 1.9	31.5 ± 3.7
	50	85.4 ± 1.3	35.2 ± 2.1	41.2 ± 1.9
	100	76.4 ± 1.7	39.6 ± 1.3	51.84 ± 2.7

Data are represented as mean ± SEM. EAC, ethyl acetate fraction of *Chrozophora tinctoria*; DCMC, dichloromethane fraction of *Chrozophora tinctoria*; DW, distilled water; PO, per orally/by orally; %, percentage.

## Data Availability

The data such as the source files associated with these findings are available from the corresponding author upon request.

## Supplementary Materials

The following supporting information can be downloaded at: https://www.mdpi.com/article/10.3390/molecules27072143/s1, Supplementary Table S1: Chemical structure of the compounds identified in DCMC and EAC fractions.

## Sample Availability

Samples of the fractions are available from the author (Ayaz Ali Sher).
