# Supplementary material for: Prokinetic and Laxative Effects of Chrozophora tinctoria Whole Plant Extract
Source: Molecules. 2022 Mar 26;27(7):2143. doi: 10.3390/molecules27072143 (PMC9000247; doi:10.3390/molecules27072143)
Supplement: Supplementary file 1 [file molecules-27-02143-s001.zip › molecules-1587755-supplementary.pdf]

Supplementary Materials

Supplementary Table S1. Chemical structure of the compounds identified in DCMC and EAC fractions.

Structures of compounds of DCMC of *Chrozophora tinctoria*		
**S.No**	**Compound name**	**Structure**
**1**	Hydroperoxide, 1-ethyl butyl	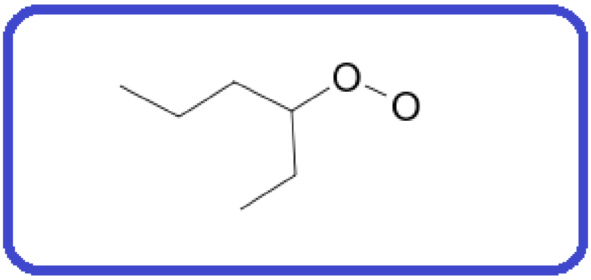
**2**	Silane, chlorodiisopropylmethyl-	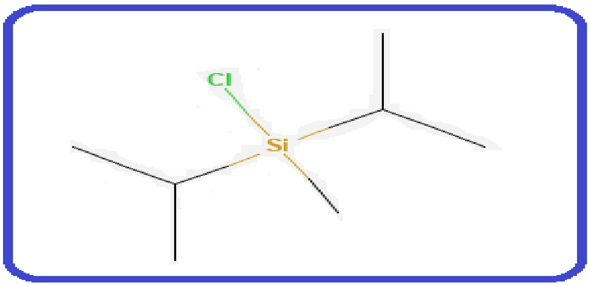
**3**	1-Hexadecene	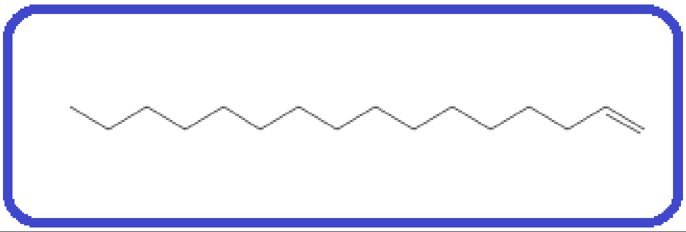
**4**	3,7,11,15-Tetramethyl-2-hexadecen-1-ol	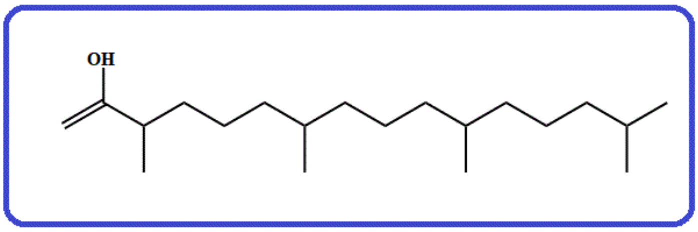

**5**	Hexadecanoic acid, methyl ester	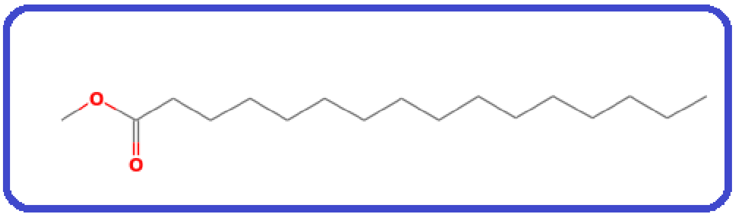
**6**	1-Eicosanol	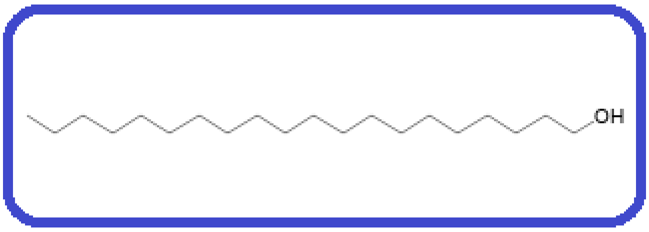
**7**	10-Octadecanoic acid, methyl ester	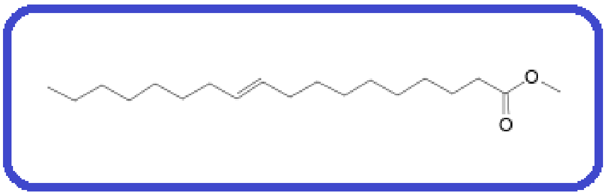
**8**	9,12,15-Octadecatrienoic acid, 2,3-bis[(trimethylsilyl)oxy]propyl ester, (Z,Z,Z)-	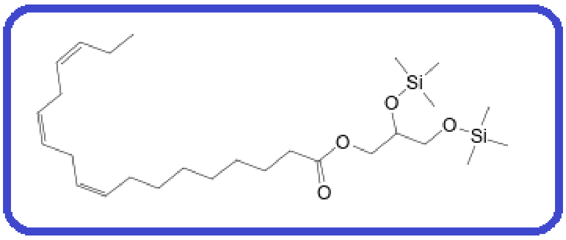
**9**	17-Pentatriacontene	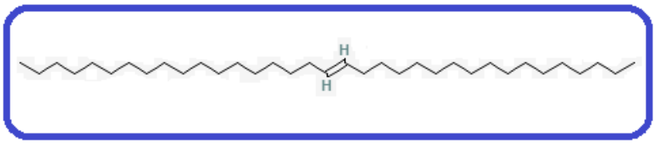
**10**	1,2-Benzenedicarboxylic acid, diisooctyl ester	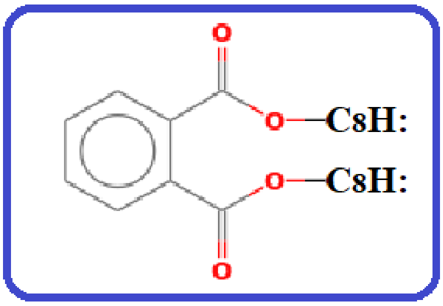

Structures of compounds of EAC of *Chrozophora tinctoria*		
**S.No**	**Compound name**	**Structure**
**1**	Imipramine	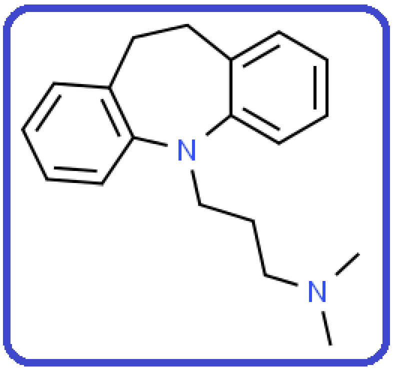
**2**	Hexadecanoic acid, methyl ester	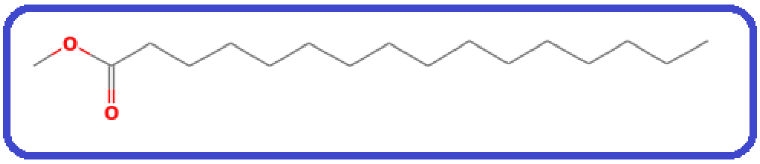
**3**	3,7,11,15-Tetramethyl-2-hexadecen-1-ol	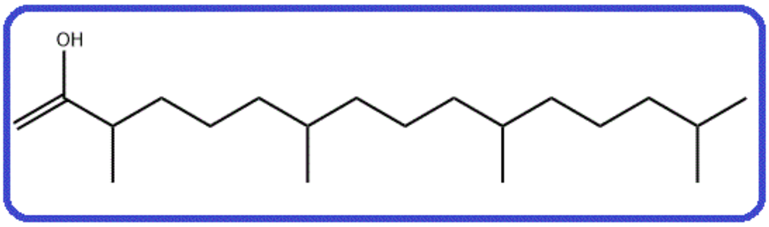
**4**	Pentadecanoic acid, 14-methyl-, methyl ester	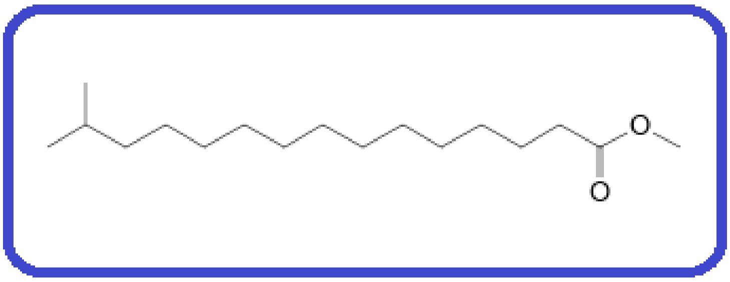

**5**	Hexadecanoic acid, ethyl ester	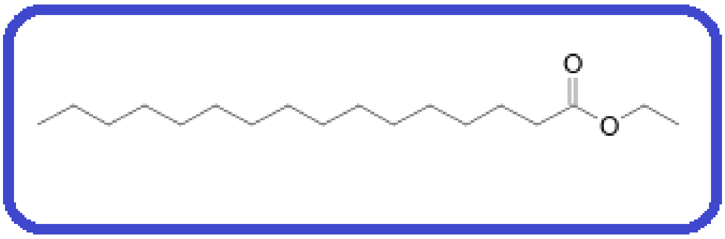
**6**	Nonanoic acid, 9-(0-propylphenyl)-methyl ester	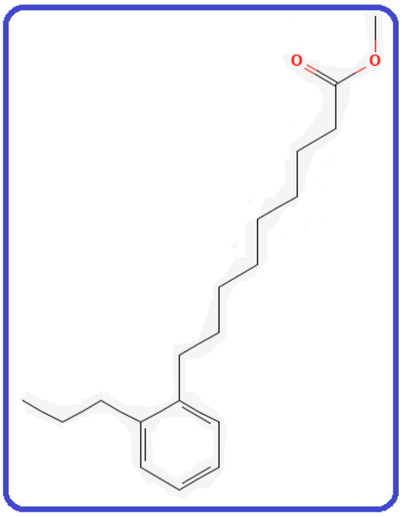
**7**	9,12,15-Octadecatrienoic acid, ethyl ester, (Z,Z,Z)-	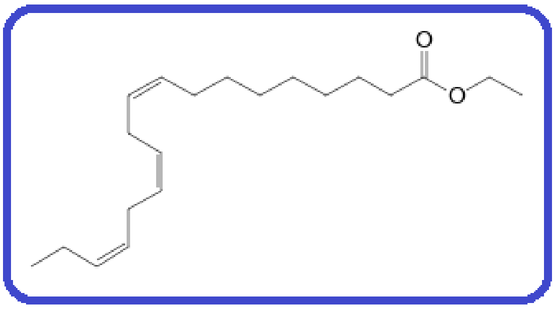
